# Immunohistochemical Expression of FOXP3+ Regulatory T Cells in Proteinuric Primary Glomerulopathies

**DOI:** 10.1155/2021/9961713

**Published:** 2021-07-10

**Authors:** Georgios Vlachopanos, Argyrios Georgalis, Pinelopi Korkolopoulou, Efstratios Patsouris, Harikleia Gakiopoulou

**Affiliations:** ^1^Department of Nephrology, General Hospital of Nikea, Nikea 18454, Greece; ^2^First Department of Pathology, School of Medicine, National and Kapodistrian University of Athens, Athens 11527, Greece; ^3^Clinic for Transplantation Immunology and Nephrology, University Hospital Basel, Basel 4031, Switzerland

## Abstract

FOXP3+ regulatory T-cell (Tregs) detection in renal allograft biopsies has been associated with a less intense immune response. Data about FOXP3+ Tregs' presence and role in primary glomerulopathies of native kidneys are minimal. We comparatively studied the immunohistochemical expression of FOXP3+ Tregs, CD4+ and CD3+ T cells in IgA nephropathy (IgAN), focal segmental glomerulosclerosis (FSGS), and membranous glomerulopathy (MGN). We retrospectively reviewed 71 renal biopsies (28 from patients with IgAN, 22 from patients with FSGS and 21 from patients with MGN) performed with proteinuria as the main indication. FOXP3+ Tregs and CD4+ and CD3+ T cells in inflammatory cell infiltrates of the interstitial tissue and periglomerular space were automatically counted using image analysis software. Univariable and multivariable logistic regressions were applied for statistical analysis. Nuclear FOXP3+ immunohistochemical expression was observed in T cells in 64% of IgAN cases, 77% of FSGS cases, and 76% of MGN cases (*p* > 0.05). Absolute FOXP3+ Tregs count in the interstitial tissue was higher in patients without arteriolar hyalinosis than in those with arteriolar hyalinosis (1.814 ± 2.160 vs. 831 ± 696; *p* = 0.029). In patients with a high FOXP3+/CD4+ ratio in the interstitial tissue, the odds ratio for CKD-EPI eGFR ≥60 ml/min/1.73 m^2^ at biopsy was 4.80 (95% CI: 1.29–17.91; *p* = 0.019). FOXP3+ Tregs intrarenal infiltration in primary glomerulopathies is common. FOXP3+ Tregs' increased expression may be associated with milder histological lesions. High FOXP3+/CD4+ ratio in the interstitial tissue may have prognostic significance for renal function preservation.

## 1. Introduction

The theory of T-cell immunoregulatory action emerged in the early 1970s, following the discovery of lymphocyte subsets and the detection of their ability to suppress the immunological response against specific antigens [1]. Regulatory T cells (Tregs) are CD4+ cells that express the CD25 surface marker [2]. The balance between Tregs and effector T cells is necessary for the maintenance of immune tolerance and immune system homeostasis. Two forms of Tregs have been identified: (1) natural Tregs (nTregs), which are produced in the thymus, and (2) induced Tregs (iTregs), which originate from the activation of naive T cells in the presence of TGF-*β* [3, 4]. The transcription factor forkhead box P3 (FOXP3) is required for Tregs development and function. It was discovered in 2003 and constitutes the most specific Tregs marker to date [5, 6]. Although FOXP3 expression in mice remains exclusively restricted to Tregs, it may be transiently induced in human activated effector T cells that lack regulatory properties [7]. Two FOXP3 isoforms have been described in human T cells. They are discriminated functionally by their difference in the capacity to: (1) bind to the nuclear receptor ROR*γ*T (retinoic acid receptor-related orphan receptor *γ*) leading to inhibition of the Th17 cell response and (2) permit expansion of Tregs in the presence of rapamycin [8].

Tissue expression of FOXP3+ Tregs has been extensively studied in the clinical setting of the acute rejection of renal allografts. Some studies have published promising results linking FOXP3+ Tregs expression with a decrease in immunological lesions and a favorable prognosis. More specifically, increased infiltration by FOXP3+ Tregs was associated with reduced interstitial inflammation and better allograft function [9, 10]. However, other studies failed to detect a protective effect of FOXP3+ Tregs in acute rejection [11]. It has been postulated that FOXP3+ Tregs enrichment inside heavily inflamed allografts tissue does not signal an alloimmune response, which is directed towards tolerance, but it rather reflects an immune injury evolving to chronicity [12]. As a result, FOXP3+ Tregs assessment should be performed cautiously in case of an overwhelming inflammatory process, and the ratio of FOXP3+ Tregs to total CD4+ or CD3+ T cells may be more informative than their absolute number. The most tangible evidence of the beneficial effect of FOXP3+ Tregs comes from their use for therapeutic purposes. FOXP3+ Tregs ex vivo expansion and perioperative administration achieved long-term tolerance (without the need for immunosuppressive drugs) in mice which were subjected to irradiation, followed by bone marrow and cardiac transplantation. FOXP3+ Tregs therapeutic potential is currently tested in clinical trials [13, 14].

Although data about the contribution of FOXP3+ Tregs in the pathogenesis of primary glomerulopathies and systematic autoimmune diseases of native kidneys are scarce, they imply that endogenous FOXP3+ Tregs reduction may negatively impact renal injury progression. FOXP3+ Tregs depletion led to the aggravation of histological lesions in murine crescentic glomerulonephritis [15]. FOXP3 mRNA levels in peripheral blood mononuclear cells of children with active Henoch–Schönlein purpura were significantly less than healthy controls [16]. We hypothesized that FΟΧP3+ Tregs are actively involved in the modification of renal tissue injury in primary glomerulopathies, and we conducted the present study that aims at: (1) describing the prevalence of FΟΧP3+ Tregs in the intrarenal infiltrates of inflammatory cells in IgA nephropathy (IgAN), focal segmental glomerulosclerosis (FSGS), and membranous glomerulopathy (MGN); (2) evaluating whether the presence of FΟΧP3+ Tregs is associated with histological lesions and clinical outcome measures such as renal function and proteinuria; and (3) correlating automated counting of FOXP3+ Tregs by image analysis software with conventional assessment by an independent pathologist.

## 2. Patients and Methods

### 2.1. Patients

We retrospectively reviewed tissue samples from the renal biopsies of 71 patients with primary glomerulopathies, which were performed between May 2009 and June 2015. The nature of our study was retrospective as biopsies were performed in the past in different referring hospitals, and renal tissue samples were dispatched to our central pathology department for examination. Demographic, clinical, and laboratory patient data were recorded at the time of renal biopsy in the referring hospitals and communicated to us; the study design did not include prospective patient follow-up. Therefore, we correlated histologic findings with simultaneous patient data. Proteinuria was the main indication for performing renal biopsy; renal function was stable in all patients, and no recent episodes of acute kidney injury were documented. The cohort of 71 patients included 28 cases of IgA nephropathy (IgAN), 22 cases of focal segmental glomerulosclerosis (FSGS), and 21 cases of membranous glomerulopathy (MGN). Histological diagnosis was based on the findings of light, immunohistochemistry, and/or electron microscopy. Causes of secondary glomerulopathies in examined tissue samples had been excluded by the appropriate clinical and laboratory workup.

Biopsy specimens were fixed in buffered formalin and embedded in paraffin. Twelve to twenty serial sections cut at 2 to 4 *μ*m were available in each sample for hematoxylin-eosin and conventional histochemical stains (PAS, Masson, silver, and Congo red). IgAN cases were classified according to the Oxford classification [17] and FSGS cases according to the Columbia classification [18], whereas MGN cases were staged according to Churg and Ehrenreich [19]. Standard histological lesions in each nephron segment (glomerulus, vasculature, interstitial tissue, and tubules) were assessed qualitatively (presence or not) except for: (1) percentage of segmentally or globally sclerosed glomeruli over total glomeruli number in each case, (2) percentage of glomeruli with crescents (when available) over total glomeruli number in each case, and (3) interstitial fibrosis/tubular atrophy and interstitial inflammation, which were semiquantitatively assessed according to the extent (<25%, ≥25%–<50%, and ≥50%) and degree (absent, sparse, and dense) respectively.

### 2.2. Immunohistochemistry

Immunohistochemical examination of all samples was done on consecutive, formalin-fixed, paraffin-embedded sections 4 *μ*m thick, using a two-step technique (two-step peroxidase-conjugated polymer technique (DAKO EnVision kit, DAKO, Carpinteria, CA)]. Slides were heated at 37°C and deparaffinized and rehydrated through a graded series of ethanols. Subsequently, the slides were pretreated with a citrate buffer solution at pH 6, in a microwave oven for 20 minutes. A monoclonal primary antibody against FOXP3 was used for FOXP3+ Tregs detection at a dilution of 1:100 (236 A/E7, Abcam, UK). Monoclonal primary antibodies were also used for CD3 and CD4 surface markers detection at a dilution of 1:350 and 1:50, respectively (F7.2.38 and 4Β12, Dako, Denmark, respectively). Archival paraffin blocks containing lymphoid tissue from healthy controls undergoing adenotonsillectomy for sleep-disordered breathing were used as positive controls in order to test primary antibodies (informed consent had been received). Sections with no primary antibody were used as negative controls.

### 2.3. Morphometric Image Analysis

Images of immunohistochemically stained sections were captured with a Nikon DS-2 MV color CCD digital camera mounted on a Nikon Eclipse 80i microscope (Nikon Co., Tokyo, Japan) using *a* × 400 objective and stored as TIFF (tagged image file format) files. The aim was to assess quantitatively the presence of FOXP3+ Tregs, and CD4+ and CD3+ Τ-cells in the interstitial tissue and in the periglomerular space; these cells were only qualitatively (presence or not) assessed inside glomeruli and between epithelial cells of the urinary tubules because they were rarely observed at these locations. We defined as periglomerular space the area around each glomerulus as thick as 50% of the glomerular diameter. Infiltrates of inflammatory cells were photographed initially at hotspots of increased expression and, then, in consecutive sections (up to 10 maximum), in order to calculate the average cell number in each infiltrate. Counting of FOXP3+ Tregs, and CD4+ and CD3+ Τ-cells was done automatically in digitally stored photographs using image analysis software Image-Pro Plus (version 5.1.2.59, Media Cybernetics, USA). The distinction between positively stained (dark brown) and negatively stained (blue) cells was done using a prespecified hue threshold in image analysis software, which was kept unchanged throughout the study period. Absolute count of FOXP3+ Tregs per mm^2^ and their ratio to CD4+ and CD3+ T cells were correlated with histological parameters and with clinical outcome measures (proteinuria, estimated glomerular filtration rate with the CKD-EPI equation (CKD-EPI eGFR)). Finally, FOXP3+ Tregs, and CD4+ and CD3+ T cells expression was also assessed by an independent pathologist (who was blinded to the results of automated counting with image analysis software), scored in a scale from 0 to ++++ and compared to automated counting.

### 2.4. Statistical Analysis

Data are presented as mean value ± standard deviation or as percentage. For continuous variables, analysis of variance (ANOVA) and *t*-test were used for between-group comparisons. For categorical variables, the *x*^2^ test and Fisher's exact test were used, as appropriate. The Bonferroni correction was applied in case of multiple comparisons. The association between outcome measures and FOXP3+ Tregs was explored through logistic regression models. The correlation between automated counting of FOXP3+ Tregs and assessment by an independent pathologist was done using Pearson's *r* correlation coefficient. The level of statistical significance was set at *p* < 0.05. Statistical analysis was performed with IBM SPSS Statistics for Windows (version 22.0, IBM Corp., Armonk, NY).

## 3. Results

Patient characteristics are displayed in Table 1. MGN patients were older than those with FSGS (56 ± 13 vs. 42 ± 20 years respectively; *p* = 0.015). IgAN patients manifested lower proteinuria compared with FSGS and MGN patients (2.0 ± 1.9 g/day vs. 5.3 ± 4.2 g/day vs. 6.7 ± 2.8 g/day, respectively; *p* = 0.001 and *p* < 0.001) and accordingly lower rates of the nephrotic syndrome but had greater hematuria rate than the other two glomerulopathies (93% vs. 55% in FSGS and 71% in MGN; *p* = 0.001 and *p* = 0.006, respectively). No statistically significant difference was found regarding gender, age, renal function level (eGFR calculated with the CKD-EPI equation), and arterial hypertension.

**Figure 1:**
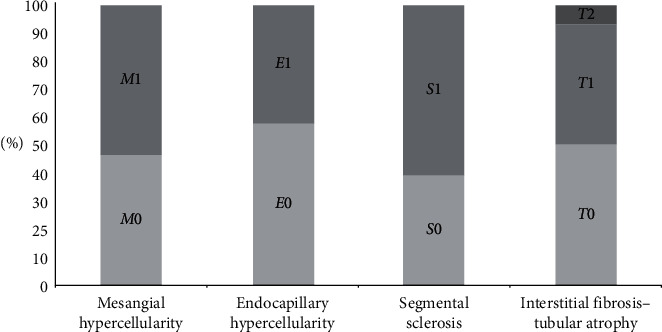
Stacked bar chart of Oxford classification parameter frequencies in patients with IgA nephropathy. Mesangial hypercellularity was almost equally scored as *M*0 (<50% of glomeruli showing mesangial hypercellularity) and *M*1 (>50% of glomeruli showing mesangial hypercellularity); endocapillary hypercellularity was more frequently scored as *E*0 (no endocapillary hypercellularity) than *E*1 (any glomeruli showing endocapillary hypercellularity); absence of segmental sclerosis (*S*0) was observed less than the presence of segmental sclerosis (*S*1); and 50% of patients showed interstitial fibrosis 0–25% (*T0*), 43% had interstitial fibrosis 26–50% (*T*1), and only 7% had interstitial fibrosis >50% (*T*2).

Frequencies of the histological parameters that are included in the Oxford classification of IgAN are shown in Figure 1: mesangial hypercellularity was scored as *M*0 (<50% of glomeruli showing mesangial hypercellularity) in 13 patients (46%) and *M*1 (>50% of glomeruli showing mesangial hypercellularity) in 15 patients (54%); endocapillary hypercellularity was scored as *E*0 (no endocapillary hypercellularity) in 16 patients (57%) and *E*1 (any glomeruli showing endocapillary hypercellularity) in 12 patients (43%); 11 patients (39%) did not have segmental sclerosis (*S*0) whereas 17 patients (61%) had segmental sclerosis (*S*1); and 14 patients (50%) showed interstitial fibrosis 0–25% (*T*0), 12 patients (43%) had interstitial fibrosis 26–50% (*T*1), and 2 patients (7%) had interstitial fibrosis >50% (*T*2).

In patients with FSGS, the tip lesion variant was present in 3 patients (14%); the cellular variant was also present in 3 patients (14%); the perihilar variant was present in 8 patients (36%); and the not-otherwise-specified (NOS) lesion was present in 8 patients (36%). In MGN patients, 14 patients (67%) had stage ΙΙ glomerulopathy, and 7 patients (33%) had stage ΙΙΙ glomerulopathy according to Churg and Ehrenreich. Light microscopy standard histologic lesions are shown in Table 2: FSGS patients had the higher proportion of glomeruli with segmental sclerosis (16% ± 9%) followed by IgAN patients (10% ± 9%) and MGN patients (3% ± 5%). FSGS patients also showed a significantly higher proportion of glomeruli with global glomerulosclerosis compared with MGN patients (33% ± 24% and 13% ± 13%, respectively; *p* < 0.001). There was no statistically significant difference between the three glomerulopathies in the extent of interstitial fibrosis-tubular atrophy, the degree of interstitial inflammation, and the presence of arterial hyalinosis. Interstitial inflammation was not observed in 30% of total patients. In the rest of our cohort, interstitial inflammation was located in areas of interstitial fibrosis in 61% of the patients, and in only 9% of the patients, interstitial inflammation was extended beyond areas of interstitial fibrosis (no statistical difference between the three glomerulopathies). Fibrous and fibrocellular crescents were observed only in IgAN patients (21% of them), but the proportion of glomeruli with crescents over the total glomeruli number was rather low (3% ± 7%).

**Figure 2:**
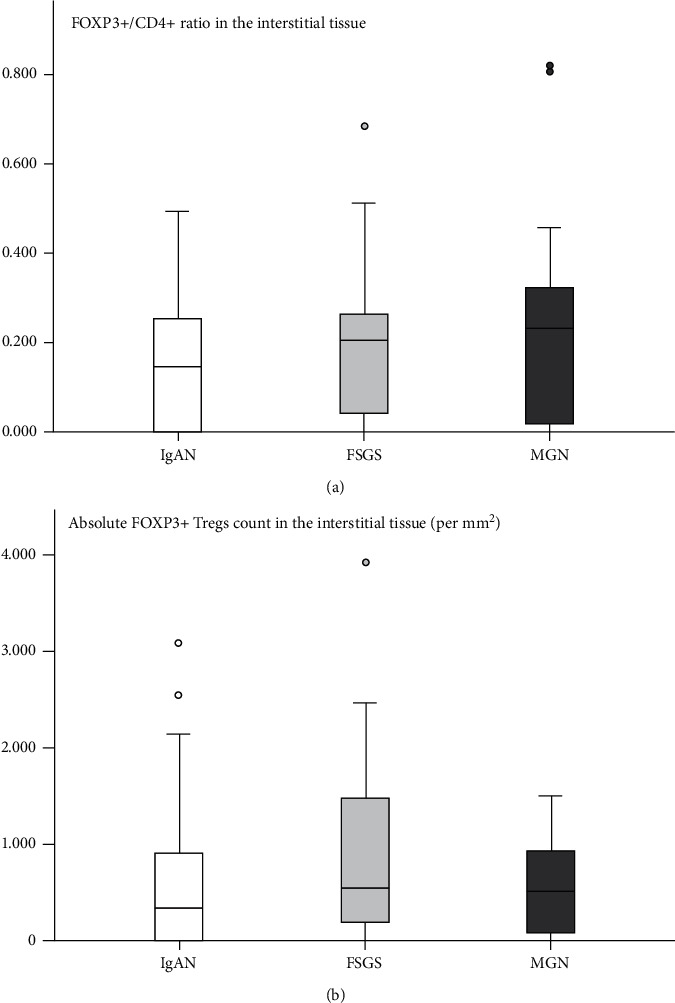
FOXP3+ Tregs detection inside inflammatory cell infiltrates was frequent. They were observed in the interstitial tissue in 64% of patients with IgAN, 77% of patients with FSGS, and 76% of patients with MGN. (a) Comparative diagram of FOXP3+/CD4+ ratio in the interstitial tissue (*p* value >0.05 for all comparisons). No statistical difference was found between the three glomerulopathies in FOXP3+ Tregs over the CD4+ ratio. (b) Comparative diagram of absolute FOXP3+ Tregs count in the interstitial tissue (per mm^2^; *p* value >0.05 for all comparisons). No statistical difference was found between the three glomerulopathies in absolute FOXP3+ Tregs count. FSGS: focal segmental glomerulosclerosis, IgAN: IgA nephropathy, MGN: membranous glomerulopathy, and Tregs: regulatory T cells.

**Figure 3:**
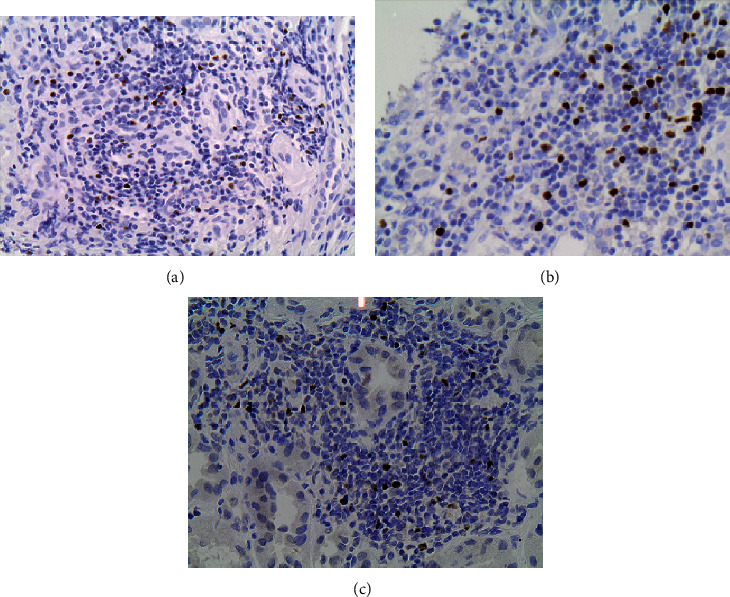
Immunohistochemical stain of FOXP3+ regulatory T cells (Tregs) in interstitial inflammatory cell infiltrates in: (a) IgA nephropathy (IgAN), (b) focal segmental glomerulosclerosis (FSGS), and (c) membranous glomerulopathy (MGN). Cells stained dark brown are considered positively stained FOXP3+ Tregs, and blue-stained cells are negatively stained lymphocytes.

**Figure 4:**
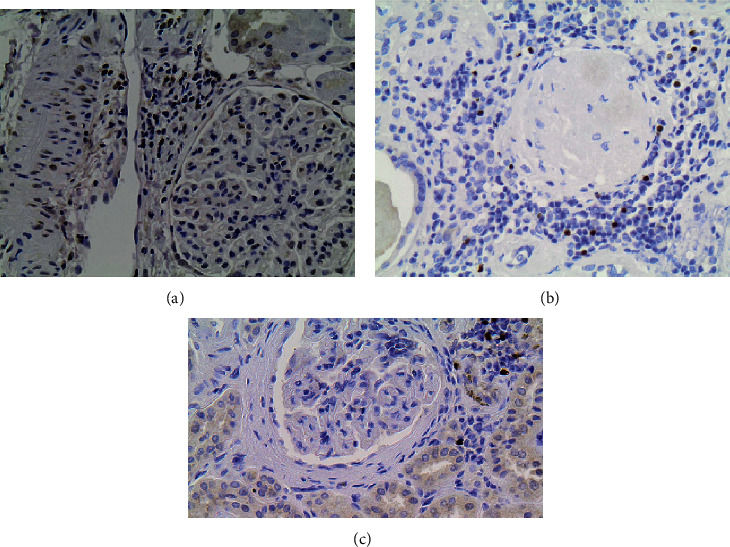
Immunohistochemical stain of FOXP3+ regulatory T cells (Tregs) in periglomerular space inflammatory cell infiltrates in: (a) IgA nephropathy (IgAN), (b) focal segmental glomerulosclerosis (FSGS), and (c) membranous glomerulopathy (MGN). Cells stained dark brown are considered positively stained FOXP3+ Tregs, and blue-stained cells are negatively stained lymphocytes.

FOXP3+ Tregs detection inside inflammatory cell infiltrates was frequent. FOXP3+ Tregs in the interstitial tissue were observed in 64% of patients with IgAN, 77% of patients with FSGS, and 76% of patients with MGN (*p* > 0.05). No statistical difference was found between the three glomerulopathies in the absolute count of FOXP3+ Tregs in the interstitial tissue per mm^2^ and FOXP3+ Tregs over CD4+ and CD3+ ratios (Figure 2; Table 3). FOXP3+/CD4+ ratio was higher than the FOXP3+/CD3+ ratio, and both ratios were higher in the interstitial tissue than in periglomerular space. FOXP3+ Tregs were rarely observed inside glomeruli (in 6% of total patients) and between tubular cells (in 25% of total patients). Immunohistochemically stained images of interstitial tissue and periglomerular space that were used for FOXP3+ Tregs automated counting are illustrated in Figures 3 and 4, respectively.

The association between absolute FOXP3+ Tregs count in the interstitial tissue and light microscopy histological lesions is described in Table 4. FOXP3+ Tregs count in the interstitial tissue was higher in patients without arterial hyalinosis than in those with arterial hyalinosis (1.814 ± 2.160 per mm^2^ vs. 831 ± 696; *p* = 0.029). A similar trend was observed for segmental sclerosis, but it did not attain statistical significance (1.477 ± 2.315 FOXP3+ Tregs per mm^2^ in patients without segmental sclerosis vs. 1.047 ± 858 in patients with segmental sclerosis, *p* > 0.05). In patients with global glomerulosclerosis, interstitial fibrosis ≥25%, or dense interstitial inflammation, FOXP3+ Tregs count in the interstitial tissue was not significantly different. All comparisons regarding FOXP3+ Tregs in the periglomerular space were not significantly different as well.

In patients with FOXP3+ Tregs infiltrates in the interstitial tissue and high FOXP3+/CD4+ ratio, the odds ratio for CKD-EPI eGFR ≥60 ml/min/1.73 m^2^ at biopsy was 4.80 (95% CI: 1.29–17.91; *p* = 0.019) with the use of multivariable logistic regression (Table 5). We defined it as a high FOXP3+/CD4+ ratio, values above the average (=0.185). The final multivariable model was adjusted for the following confounding factors: age, gender, and interstitial fibrosis. We did not find a statistically significant association between high FOXP3+/CD4+ ratio and proteinuria >3.5 g/day at biopsy (odds ratio: 0.93; 95% CI: 0.29–3.00; *p* = 0.902).

**Figure 5:**
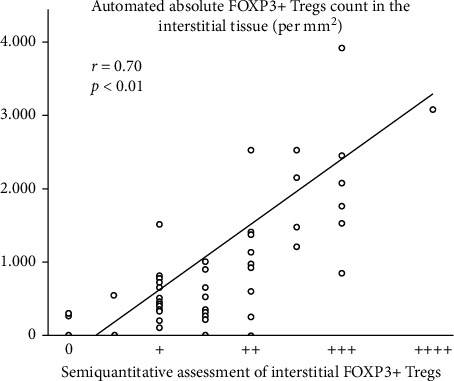
Line diagram of the correlation between interstitial FOXP3+ Tregs automated counting with image analysis software and semiquantitative assessment by an independent pathologist (in a scale from 0 to ++++). Correlation coefficient *r* was 0.70 (*p* < 0.01) between the two methods denoting satisfying agreement. *r*: Pearson's correlation coefficient and Tregs: regulatory T cells.

The correlation between interstitial FOXP3+ Tregs automated counting with image analysis software and semiquantitative assessment by an independent pathologist on a scale from 0 to ++++ is shown in Figure 5. Pearson's correlation coefficient *r* was 0.70 (*p* < 0.01). Moreover, equally significant was a correlation between periglomerular FOXP3+ Tregs automated counting and semiquantitative pathologist assessment. Hence, the agreement between these two methods is deemed to be satisfying.

## 4. Discussion

This is a comparative study on FOXP3+ Tregs renal tissue expression in human adults with the primary glomerulopathies IgAN, FSGS, and MGN. We demonstrated that FOXP3+ Tregs often participate to a significant degree in the inflammatory infiltrates composition in these diseases, and we correlated increased FOXP3+ Tregs renal tissue expression with milder histological lesions. In addition, we found that increased interstitial FOXP3+/CD4+ ratio seems to be associated with a better prognosis regarding renal function.

Leading theories about IgAN, FSGS, and MGN pathogenesis generally ignore FOXP3+ Tregs potential role on renal injury modulation. IgAN is caused by aberrant glycosylation of IgA, formation of autoantibodies against IgA, and ultimately, deposition of immunocomplexes inside glomeruli [20], while in MGN, the formation of immunocomplexes in glomerular basement membrane is caused by autoantibodies directed against *in situ* antigens [21]. Effacement of podocyte foot processes is critical for FSGS pathogenesis; the structure and biological action of the circulating permeability factor have not been elucidated yet [22]. However, we believe that FOXP3+ Tregs detection in primary glomerulopathies is of special importance and not a mere epiphenomenon lacking clinical significance. FOXP3+ Tregs are virtually nonexistent in healthy renal and extrarenal tissue; our findings of FOXP3+ Tregs' presence in glomerulopathic tissue strongly denote activation of immunological pathways [23, 24]. Moreover, our results are in agreement with those of prior studies: In a study of 38 children with idiopathic nephrotic syndrome (FSGS included) and in a study of 63 adult patients with IgAN, it was found that FOXP3+ Tregs renal tissue expression was rather common [25, 26]. Recently, Chebotareva et al. reported that a significant decrease in the number of Tregs in the renal tissue is revealed in patients with chronic glomerulonephritis and nephrotic syndrome versus those with chronic glomerulonephritis without nephrotic syndrome [27, 28]. It is our belief that further research is required to delineate factors and mechanisms that regulate intrarenal expression of FOXP3+ Tregs.

The frequent occurrence of FOXP3+ Tregs in fibrotic areas of interstitial tissue leads to the question of whether the development of chronic kidney disease (CKD) and chronic interstitial fibrosis (independent of the initial immunological cause) precedes FOXP3+ Tregs' presentation. There is evidence that FOXP3+ Tregs are involved in CKD progression [29, 30]. However, we also identified inflammatory infiltrates containing FOXP3+ Tregs in interstitial tissue areas without fibrosis. Furthermore, there were patients in our cohort with a normal or quasi-normal renal function who demonstrated FOXP3+ Tregs intrarenal infiltrates (54% of our patients had a CKD-EPI eGFR ≥ 60 ml/min/1.73 m^2^ and 18% had a CKD-EPI eGFR ≥ 90 ml/min/1.73 m^2^). Thus, given the heterogeneity of our patient cohort regarding renal function level, we consider that FOXP3+ Tregs recruitment in renal tissue may occur early in the pathogenesis of glomerulopathies before the establishment of severe histological lesions.

The use of immunohistochemistry for the evaluation of FOXP3+ Tregs tissue expression and image analysis software for automated cell counting are significant strengths of our study. According to literature, immunohistochemistry is the most commonly used method for assessing FOXP3+ Tregs in tissue samples along with FOXP3 mRNA measurement using polymerase chain reaction (PCR). However, immunohistochemistry is more effective compared with FOXP3 mRNA measurement for bona fide (genuine) FOXP3+ Tregs detection because it easily identifies cells that overexpress the FOXP3 marker and that are more likely to be bona fide FOXP3+ Tregs [9]. On the contrary, FOXP3 mRNA measurement cannot distinguish between cells with increased or reduced FOXP3 marker expression, and therefore, it may erroneously identify activated effector T cells lacking immunoregulatory activity along with bona fide FOXP3+ Tregs [31]. Semiquantitative FOXP3+ Tregs assessment by an independent pathologist blinded to the results of automated cell counting showed that these two methods significantly correlate and that automated FOXP3+ Tregs counting can be extensively used in future research since it is a reliable and convenient method.

Although it is universally accepted that FOXP3+ Tregs biological action aims at suppressing the immune response, the association between the FOXP3+ Tregs immunohistochemical expression and standard histological lesions seems to be controversial in primary as well as in secondary glomerulopathies. This means that isolated assessment of FOXP3+ Tregs tissue expression may not be adequate to highlight potential protective properties. In our analysis, patients without arteriolar hyalinosis had higher absolute FOXP3+ Tregs count in interstitial tissue compared to patients with arteriolar hyalinosis (possibly because milder disease course in patients with higher absolute FOXP3+ Tregs count is associated with better blood pressure control [32]). Afeltra et al. reported that FOXP3+/CD3+ ratio in interstitial tissue was low in patients with systemic lupus erythematosus (SLE) nephritis class IV and high activity index [33]. In contrast, Lin et al. found that absolute FOXP3+ Tregs count in interstitial tissue was significantly higher in patients with IgAN and interstitial fibrosis >25% [26]. Some authors propose that interleukin 17 expression should be assessed along with FOXP3+ Tregs expression (in order to compare balance between Th1 immune response, which is mediated by FOXP3+ Tregs, and Th17 immune response) or that TGF-*β* and interleukin 10 expression should be simultaneously assessed because FOXP3+ Tregs modulatory action is mediated by these cytokines [34, 35]; their lack is a limitation of our study. However, the use of FOXP3+/CD4+ and FOXP3+/CD3+ ratios for the evaluation of FOXP3+ Tregs tissue expression is considered the optimal method in general because it reflects the association between the regulatory arm and the effector arm of the immune response [36].

The finding that high FOXP3+/CD4+ ratio in the interstitial tissue is associated with the better renal function (CKD-EPI eGFR ≥ 60 ml/min/1.73 m^2^) at biopsy after adjustment for confounding factors such as age, gender, and interstitial fibrosis may have important prognostic value. However, that should be interpreted with caution because the present study lacks patient follow-up after biopsy, and as such, it cannot prove an etiologic association between these two variables. We believe that further prospective studies examining longitudinal FOXP3+ Tregs tissue expression in conjunction with GFR trajectory are required to confirm this association. Future research should also be directed towards the therapeutic use of FOXP3+ Tregs. Purified CD4+CD25+ Tregs transfer to rats with an idiopathic nephrotic syndrome model resulted in significant proteinuria reduction [37]. Ongoing clinical trials with FOXP3+ Tregs in the field of kidney transplantation could provide useful hindsight for the therapeutic application of FOXP3+ Tregs in primary glomerulopathies and systematic autoimmune diseases of native kidneys.

## 5. Conclusions

We showed that FOXP3+ Tregs are consistently expressed in the renal tissue of human adults with proteinuric primary glomerulopathies such as IgAN, FSGS, and MGN. We found that in patients without arteriolar hyalinosis, the absolute FOXP3+ Tregs count in interstitial tissue was significantly higher and that high FOXP3+/CD4+ ratio is significantly associated with CKD-EPI eGFR ≥60 ml/min/1.73 m^2^ at biopsy. These results imply that FOXP3+ Tregs may favorably modify glomerulopathy progression and that they may have potential diagnostic and therapeutic implications.

## Figures and Tables

**Table 1:** Demographic, clinical, and laboratory patient characteristics.

	Total (Ν = 71)	IgAN (Ν = 28)	FSGS (Ν = 22)	MGN (Ν = 21)	*p* value
Age (years)	48 ± 17	47 ± 14	42 ± 20_a_	56 ± 13_a_	a: 0.015
Gender (male, %)	63	50	68	76	ns
CKD-EPI eGFR (ml/min/1.73 m^2^)	61 ± 32	59 ± 29	55 ± 31	70 ± 35	ns
Proteinuria (g/day)	4.5 ± 3.6	2.0 ± 1.9_a,b_	5.3 ± 4.2_a_	6.7 ± 2.8_b_	a: 0.001 b: <0.001
Nephrotic syndrome (%)	52	20	60	81	a: 0.001 b: <0.001
Arterial hypertension (%)	37	29	41	43	ns
Glomerular hematuria (%)	75	9_*β*a,b_	55_a_	71_b_	a: 0.002 b: 0.006

*Note.* Data are presented as mean value ± standard deviation or as percentage. FSGS: focal segmental glomerulosclerosis, IgAN: IgA nephropathy, CKD-EPI eGFR: glomerular filtration rate estimated with the CKD-EPI equation, MGN: membranous glomerulopathy, and ns: not significant.

**Table 2:** Light microscopy histological lesions.

		Total (*N* = 71)	IgAN (*N* = 28)	FSGS (*N* = 22)	MGN (*N* = 21)	*p* value
Total glomeruli		20 ± 11	21 ± 10	21 ± 13	18 ± 8	ns
Patients with segmental sclerosis (%)		66	61	100	29	<0.001
*Percentage of segmentally sclerosed glomeruli over total glomeruli*		10 ± 10	10 ± 9_a,b_	16 ± 9_a,c_	3 ± 5_b,c_	a: 0.012 b: 0.003 c: <0.001
Patients with global glomerulosclerosis (%)		82	89	86	67	ns
Percentage of globally sclerosed glomeruli over total glomeruli		24 ± 21	26 ± 21	33 ± 24_*α*_	13 ± 13_*α*_	*α*: <0.001

*Interstitial fibrosis-tubular atrophy (%)*	<25	52	50	41	67	ns
	≥25–<50	38	43	36	33	ns
	≥50	10	7	23	0	ns

*Interstitial inflammation (%)*	Absent	30	25	23	43	ns
	Sparse	27	25	32	24	ns
	Dense	44	50	45	33	ns

Patients with interstitial inflammation inside fibrotic areas (%)		61	61	73	48	ns
Patients with arteriolar hyalinosis (%)		61	75	55	48	ns
Patients with crescents (%)		8	21	0	0	0.004
Percentage of glomeruli with crescents over total glomeruli		1 ± 4	3 ± 7	0	0	0.028

*Note.* Data are presented as mean value ± standard deviation or as percentage. FSGS: focal segmental glomerulosclerosis, IgAN: IgA nephropathy, MGN: membranous glomerulopathy, and ns: not significant.

**Table 3:** FOXP3+/CD4+ and FOXP3+/CD3+ ratios in the interstitial tissue and periglomerular space and qualitative assessment of FOXP3+ Tregs' presence inside glomeruli and between tubular cells.

	Total	IgAN	FSGS	MGN	*p* value
FOXP3+/CD4+ ratio in interstitial tissue	0.185 ± 0.200	0.146 ± 0.158	0.202 ± 0.192	0.213 ± 0.250	ns
FOXP3+/CD3+ ratio in interstitial tissue	0.100 ± 0.129	0.114 ± 0.136	0.081 ± 0.138	0.103 ± 0.111	ns
FOXP3+/CD4+ ratio in periglomerular space	0.099 ± 0.155	0.130 ± 0.188	0.072 ± 0.106	0.089 ± 0.155	ns
FOXP3+/CD3+ ratio in periglomerular space	0.055 ± 0.104	0.046 ± 0.082	0.054 ± 0.106	0.066 ± 0.128	ns
Patients with FOXP3+ Tregs' presence inside glomeruli (%)	6	4	5	10	ns
Patients with FOXP3+ Tregs' presence between tubular cells (%)	25	29	27	19	ns

*Note.* Data are presented as mean value ± standard deviation or as percentage. FSGS: focal segmental glomerulosclerosis, IgAN: IgA nephropathy, MGN: membranous glomerulopathy, ns: not significant, and Tregs: regulatory T cells.

**Table 4:** Association between absolute FOXP3+ Tregs count in the interstitial tissue (per mm^2^) and histological lesions.

	Absolute FOXP3+ Tregs count in the interstitial tissue (per mm^2^)		*p* value
Segmental sclerosis or not	1.047 ± 858	1.477 ± 2.315	ns
Global glomerulosclerosis or not	1.265 ± 1.548	632 ± 286	ns
Interstitial fibrosis ≥25% or interstitial fibrosis <25%	1.462 ± 1.766	718 ± 462	ns
Dense interstitial inflammation or sparse interstitial inflammation	1.476 ± 1.738	777 ± 837	ns
Arteriolar hyalinosis or not	831 ± 696	1.814 ± 2.160	0.029

*Note.* Data are presented as mean value ± standard deviation or as percentage. ns: not significant and Tregs: regulatory T cells.

**Table 5:** Association between high FOXP3+/CD4+ ratio in the interstitial tissue (above average value: 0.185) with renal function level and proteinuria at biopsy using multivariable logistic regression.

	CKD-EPI eGFR ≥60 ml/min/1.73 m^2^			Proteinuria ≥3.5 g/day		
	OR	95% CI	*p* value	OR	95% CI	*p* value
FOXP3+/CD4+ ratio in the interstitial tissue						
Low (<0.185)	1			1		
High (>0.185)	4.80	1.29–17.91	0.019	0.93	0.29–3.00	0.902

*Note.* The final multivariable model was adjusted for the following confounding factors: age, gender, and interstitial fibrosis. CI: confidence interval, CKD-EPI eGFR: glomerular filtration rate estimated with the CKD-EPI equation, and OR: odds ratio.

## Data Availability

The data used to support the findings of this study are available from the corresponding author upon request.
